# The Mediating Effect of Coping Style on Physical Activity and Negative Affect Caused by Public Health Emergencies: Evidence from Chinese College Students

**DOI:** 10.3390/ijerph182212086

**Published:** 2021-11-17

**Authors:** Yuetao Liu, Zhiyuan Wang, Songhui You

**Affiliations:** International College of Football, Tongji University, Shanghai 200092, China; liuyuetao@tongji.edu.cn

**Keywords:** physical activity, coping style, negative affect, public health emergency

## Abstract

In this study, we explored the relationship between physical activity (PA), coping style (CS) and negative affect caused by public health emergencies (PHENA), to examine if CS can play a mediating role between PA and PHENA, and analyzed the different effects of positive coping (PC) and negative coping (NC). Using the method of random sampling, 700 students from four universities in Beijing and Shanghai were recruited to complete questionnaires about PA, CS and PHENA. Data collection was conducted through online and offline questionnaires. Firstly, there is a significant correlation between PA, CS and PHENA. Secondly, PA can negatively predict PHENA, and PA has positive impact on PC and negative impact on NC. Thirdly, the mediating role of NC related to PHENA is significant, the mediating role of PC related to PHENA is not significant. College students’ participation in physical activity can reduce the probability of adopting negative coping mechanisms, thereby alleviating the PHENA.

## 1. Introduction

Every public health emergency (such as SARS, H1N1, Ebola) is a disaster for public health. The most recent COVID-19 pandemic has imposed enormous pressure on governments, medical and healthcare providers and the general public [1], and the pressure is still going on. The pandemic brought not only the risk of death from the viral infection but also unbearable psychological pressure to people in China and the rest of the world [2]. The physical damage caused by public health emergencies may recover in a short time, but the psychological impact will last for a long time. Public health messages often use persuasive language to change attitudes and behaviors, which can evoke a wide range of negative and positive emotional responses [3]. As COVID-19 continues, the public is experiencing different levels of psychological distress, such as nervousness, fear of infection, anxiety, depression, sleep problems, and inattention [4,5]. The college years represent a period of increased vulnerability for a wide range of mental health challenges [6]. Younger people are at especially high risk of being psychologically affected by the pandemic when they spend too much time thinking about the outbreak [7]. College students are in a special period of physical and mental development, and therefore public health emergencies can affect them psychologically in an irreversible and profound way [8].

### 1.1. Negative Affect Caused by Public Health Emergencies

Life events are an important factor in inducing negative affect, which can lead to an increase in problem behaviors and affective disorders [9]. The adverse effects created from one external crisis event (e.g., public health emergencies) will spill over and interfere with the judgment of the decision maker on other routine matters through negative affect [10]. According to research conducted by Gao during SARS, negative affect caused by public health emergencies (PHENA) includes neurasthenia, depression, obsession, anxiety, fear, and hypochondriasis [11,12]. Among them, obsession, fear, and hypochondriasis are negative affects which are unique during the special period of public health emergencies. Numerous studies have confirmed the existence of PHENA among college students. The emotional changes of college students during the SARS period are highlighted by fear [11]. During COVID-19, negative affect such as anxiety and depression are relatively common. A study of Chinese college students found that 24.9% of respondents had symptoms of anxiety, of which 3.6% were moderate or severe anxiety [13].

### 1.2. Physical Activity

Exercise intervention has long been regarded as an alternative or supplementary method for the treatment of psychological problems [14]. A wealth of evidence supports the positive impact of physical activity (PA) on mental health [15,16,17,18,19,20]. For mild-to-moderate depression, the effect of PA may be comparable to antidepressant medication and psychotherapy [21]. PA is positively associated with college students’ positive affect. PA appears to enhance positive affect during a global pandemic [22,23]. PA can also reduce the impact of anticipated negative affect [24]. Associations between PA and affect may differ depending on the extent to which one experiences stressful life events [25]. In the period of public health emergencies, whether PA can still play a role in reducing negative affect is a question worth exploring.

### 1.3. Coping Style

Coping is one of the most extensively studied concepts in psychology [26], it encompasses two concepts. The first is that of stable coping style (CS) which characterizes an individual’s interaction with his or her stressful environment, and the second involves coping skills or techniques that people use to manage specific stressful situations [27,28,29]. CS refers to the cognitive and behavioral styles used by individuals in the face of frustration and pressure [30]. Assessing an individual’s coping style and understanding its relationship with mental health is an important content of psychological research. Lazarus and Folkman proposed eight specific coping methods based on the results of factor analysis, such as confrontation, avoidance, self-control, and seeking support, etc. [31]. Stone and Neate also proposed eight coping styles, such as distraction, re-evaluation of the environment, catharsis, and relaxation [32]. Yang put forward six types of coping methods, such as selective neglect, changing the value system, recklessness or taking risks [33]. From the perspectives of different researchers, it can be seen that there are many types of coping styles. However, further analysis found that the coping styles proposed by different researchers have common characteristics, that is, some coping styles have more positive components, such as seeking support and trying to change, while others are dominated by negative components, such as avoidance and venting [34]. Many coping style assessment methods have been developed, such as the “Ways of Coping Questionnaire” compiled by Folkman and Lararus. Some scholars have tried to apply the questionnaire to the Chinese population and found that due to differences in cultural background, foreign scales are not suitable for the Chinese population. One reason is that some content is not suitable, and the other is that the results of factor analysis are inconsistent. Based on the characteristics of Chinese culture, Xie simplified and revised the foreign coping style scales, and compiled the “Simple Coping Style Questionnaire” with two dimensions, positive coping (PC) and negative coping (NC). The scale has been widely used in Chinese psychology circles. A large number of studies based on this have shown that when the NC score is high, the score for psychological problems or symptoms is also high; while when the PC score is high, the score for psychological problems or symptoms is low. Additionally, many scholars use it as an important intermediary variable for research.

### 1.4. The Present Study and Hypothetical Model

The effect of PA has been inquired in different ages of people such as children [35,36,37], adolescents [37,38,39,40], middle-aged people [41,42] and older adults [43,44,45,46]. There have also been studies on different populations, for instance, pregnant women and mothers [47,48], nurses [49], patients [50,51,52,53,54], and people with overweight/obesity [55,56]. Some scholars have also emphasized the benefits of family members exercising together, such as father–daughter [57,58] and mother–child [59]. The overwhelming majority of studies support that PA can reduce negative affect and promote mental health. The effect varies with the intensity [42] and frequency [60] of PA, age [46], gender [61], etc. A small number of studies do not support the role of PA, and they believe that both positive affect and negative affect precede rather than follow PA engagement [62].

Researches on PA during COVID-19 show that it was associated with higher resilience [46,63], less negative affect [23,37,46], and better psychological wellbeing [64,65]. Although there have been kinds studies in the literature revealing the association between PA and general negative affect such as depression and anxiety, the relationship between PA and PHENA is still unclear. 

When faced with stress or traumatic events, people will adopt different CS; some will respond positively while others negatively [40]. There is widespread conviction among healthcare professionals that coping influences affect [66]. NC may be related to psychological distress or mental illnesses such as anxiety and depression [67,68]. People with NC show a higher level of psychological distress [69]. Coping research has shown that NC, such as indulging in negative topics, can exacerbate negative affects such as anxiety [70]. In contrast, PC may promote emotional wellbeing [71]. Fredrickson believes that affect and coping can influence each other [71]. Which direction of causation is of greater importance depends on the research question, if the question is how best to intervene to reduce depression, the direction of interest is from coping to affect [72]. Coping is an important mediator in the regulation of affect [66]. Though CS is stable [73], it is not unchangeable. Many studies have confirmed that PA can affect CS [74,75,76,77]. Firstly, PA can affect people’s CS through changes in their physiological functions and mood. Secondly, through the unique cluster environment of PA, it can also affect the individual’s CS and emotional regulation [78]. To our knowledge, research on the mediating role of CS between PA and PHENA is still blank. We aim to overcome limitations of other studies that only focus on general negative affect. This study will discuss the relationship between PA and PHENA, and further try to explain its mechanism of action.

**Figure 1:**
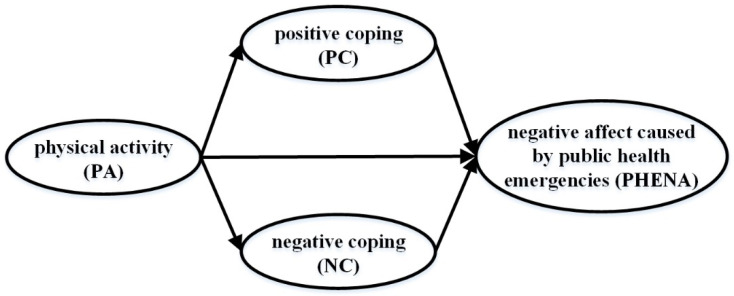
Hypothetical model.

After review of previous studies, the present study believes that CS may be an important intermediary variable between PA and PHENA. Based on this, this study takes college students as subjects and proposes the following hypotheses H1: PA is significantly negatively correlated with PHENA; H2: CS plays a mediating role between PA and PHENA. The hypothetical model is shown in Figure 1.

## 2. Materials and Methods

### 2.1. Participants and Procedure

Using the method of simple random sampling, from September to October 2020, 700 students from Renmin University of China, Beijing Sport University, Tongji University and East China Normal University were selected randomly to conduct a questionnaire survey without repeating. The four universities are different types of universities from different regions of China. We recruited respondents randomly through online chats, mailing lists and offline methods. The invitation letter contained the introduction of the research, the reward for filling out the questionnaire, the obligations of the investigator and respondent, the principle of confidentiality and the Informed Consent Form. Respondents were asked to scan a predesigned QR code to fill out the questionnaire online. Before filling out the questionnaire, the Informed Consent Form will be displayed first, and only after the content of the Informed Consent Form has been read and accepted will the respondent be allowed to enter the formal questionnaire survey. Before the start of the questionnaire survey, we carried out a test–retest reliability test with 50 samples. The interval between the two tests is 20 days, and the Pearson’s correlation coefficient is used for testing. Pearson’s correlation coefficient is used to calculate test–retest reliability on SPSS. All questionnaires are compiled, filled out and collected through Wenjuan.com. Each person can only submit the questionnaire once.

The questionnaire has 54 items in total, including three scales and some demographic variables. Bentler [79] proposed that only the ratio of sample number to item number could reach 5:1, to ensure the reliability of parameter estimation. A ratio of 10:1 can guarantee the validity of the significance test. Considering economy and sample reliability, the number of samples in this study is controlled to be between 500 and 800. We set up two screening items in the questionnaire, such as “Please choose 1 for this question”. At the same time, invalid questionnaires were eliminated according to the principle that the answering time of the questionnaires should not be less than 1 min. A total of 906 invitations were randomly distributed, of which 61 people responded and rejected the survey. When 700 samples were collected, we closed the questionnaire entry portal. A total of 700 questionnaires were received, of which 633 were valid questionnaires and therefore the effective rate was 90.43%, with no missing data. Of all valid questionnaires, 58.8% (*n* = 372) of the sample identified as female and 41.2% (*n* = 261) were male. On average, participants were 21.53 years old (SD = 2.47, Range 17–29). All participants were Chinese students from universities in Beijing and Shanghai—44.25% are undergraduate students, 49.27% are postgraduate students, and 6.48% are doctoral students.

### 2.2. Measures

#### 2.2.1. Physical Activity

The “Physical Activity Scale-3” (PARS-3) [80] is used to measure the college students’ amount of exercise from three aspects: frequency, duration and intensity. This questionnaire is the most widely used scale for measuring the amount of PA in China. Likert-5 points, ranging from 1 to 5, are used for scoring. PA intensity is measured by selecting the most suitable option from 1 (light exercise, no sweating), 2 (low intensity exercise, slightly sweating), 3 (medium-intensity, more intense and long-lasting exercise, obviously sweating), 4 (high-intensity intermittent shortness of breath exercise, sweating a lot), 5 (high-intensity continuous shortness of breath exercise, profuse sweating) according to actual situations. PA frequency is the frequency of participating in exercise. Options include 1 (1 time a month or less), 2 (2 to 3 times a month), 3 (1 to 2 times a week), 4 (3 to 5 times a week), 5 (approximately once a day). PA duration is approximately how long each exercise lasts. Options include 1 (10 min and below), 2 (11 to 20 min), 3 (21 to 30 min), 4 (31 to 59 min), 5 (60 min and above). It also surveyed the exercise programs that respondents often participated in. The test–retest reliability of the PARS-3 is 0.82, and the Cronbach α is 0.81. 

#### 2.2.2. Coping Style

The “Simple Coping Style Questionnaire” [34] is used for measurement. It includes two subscales, namely positive coping (PC) and negative coping (NC). The questionnaire consists of 20 items, involving different attitudes and measures that people may often take in daily life. Including 12 PC items such as “try to see the good side of things”, “seeking advice from relatives, friends or classmates”, and 8 NC items such as “relieving worries by smoking, drinking, taking medicine and eating”, “accept the reality, because there is no other way”, etc. The questionnaire assessed two dimensions on a 5-point Likert-type scale from 1 (never adopted) to 5 (often adopted). The test–retest reliability of the scale is 0.89. The Cronbach α of the full scale is 0.90. The PC scale is 0.89, and the NC scale is 0.78. 

#### 2.2.3. Public Health Emergency Negative Affect

The “Psychological Questionnaire for Sudden Public Health Events” [11] including five scales, namely depression, neurasthenia, fear, obsessive-anxiety and hypochondriasis is employed in the present study. In this study, 5 dimensions were combined into 4, consisting of 23 emotional reactions that may occur during the pandemic: depression-neurasthenia (11 items; e.g., “Feel annoyed and get angry easily.”), fear (4 items; e.g., “In places where people gather, especially near hospitals, feel frightened and nervous.”), obsessive-anxiety (4 items; e.g., “Wash hands repeatedly, but always feel that they are not clean enough.”), hypochondriasis (4 items; e.g., “Very concerned about any physical discomfort.”). The 23-item questionnaire assessed 4 dimensions on a 5-point Likert-type scale from 1 (none) to 5 (severe). The test–retest reliability of the questionnaire is 0.82. The Cronbach α of the questionnaire is 0.95. The depression scale is 0.96, the fear scale is 0.94, the obsessive-anxiety scale is 0.85, and the hypochondriasis scale is 0.86.

### 2.3. Data Analysis

Harman’s single-factor test method is used for principal component analysis [81,82]. A total of 7 factors with characteristic roots over 1 are generated, and the first one explains 38.44% of the variation, which is less than the critical standard of 40%. Thus, common method bias is not an issue of concern.

KMO and Barlett Test of Sphericity are used to test the data in the scales. The KMO value is 0.923, which is greater than 0.8. The *p*-value of Bartlett’s test is 0.000, which satisfies the significance test. This result confirms that the data in the scales are very suitable for factor analysis. We used SPSS to perform factor analysis to test whether the measurement items in the questionnaire are consistent with the factors we designed. By extracting principal components, 7 factors with initial eigenvalues over 1 are obtained, and the cumulative explained variance variation is 72.520%. Therefore, the explanation degree is relatively ideal. The feature value does not change significantly when more than 8 components are extracted. Therefore, 7 factors including PA, PC, NC, depression-neurasthenia, fear, obsessive-anxiety and hypochondriasis are finally extracted. The factor load of the above 7 factors corresponding to their topics is greater than 0.5, and the average variance, AVE, is greater than 0.5 as well. Among them, depression and neurasthenia are two independent factors in Gao’s research [11]. However, the concept of neurasthenia has not been universally accepted in academia. It should be noted that the term “neurasthenia” has rarely been used in clinical diagnosis by Chinese psychiatrists since the year 1995 [83]. Arthur-Kleinman believed that the disease named “neurasthenia” in China is the same as “depression” in the West. Chen Jianmei [84] believes that there is almost no essential difference between the symptoms of neurasthenia and depression. The results of this study also confirm this point. Therefore, in the present study, the factor of depression and that of neurasthenia are combined together as depression factors.

In order to test whether there is a correlation between the variables, SPSS was used for Pearson’s correlation analysis, and the mean and standard deviation were calculated. We performed SEM using AMOS 24.0.0. We established a structural equation model in AMOS based on the hypothetical model. Model fitting index χ2/df = 2.221, RMR = 0.046, GFI = 0.970, AGFI = 0.954, NFI = 0.944, RFI = 0.927, IFI = 0.968, TLI = 0.959, CFI = 0.968, RMSEA = 0.042. According to the structural equation model fitting standard proposed by Bollen [85]: χ2/df ≤ 3.00, RMSEA ≤ 0.08, CFI, NFI, RFI, IFI, AGFI ≥ 0.90. All indicators in this study have reached the fitting standard and the model is well fitted. Traditional standard procedure and bootstrap methods were adopted to test mediation.

## 3. Results

### 3.1. Descriptive Statistics and Correlations

The Pearson’s correlation analysis of the bivariate of every variable is performed in the present study. Table 1 lists the average value, standard deviation and correlation matrix of each variable. The results show that, except for PA and fear, PC and fear, PC and hypochondriasis, there is a significant correlation between the remaining variables. To be more specific, PA is positively correlated with PC while negatively correlated with NC, depression, obsessive-anxiety and hypochondriasis. Additionally, variables including NC, depression, fear, obsessive-anxiety, and hypochondriasis are all positively correlated with each other.

### 3.2. Mediating Effects of CS

To test mediation, we followed the standards steps that must be met before a variable may be considered a mediating variable. Table 2 provides the estimates obtained from the structural equation model. Model 1 shows that PA significantly predicted PHENA (β = −0.138, *p* < 0.01), supporting H1. Model 2 shows the positive impact of PA on PC (β = 0.160, *p* < 0.01) and negative impact on NC (β = −0.272, *p* < 0.001). Model 3 shows that the mediating role of NC related to PHENA (β = 0.368, *p* < 0.001) is significant, and the mediating role of PC related to PHENA (β = −0.073, *p* > 0.1) is not significant. This suggests that PA influences NC, and NC in turn influences PHENA.

**Figure 2:**
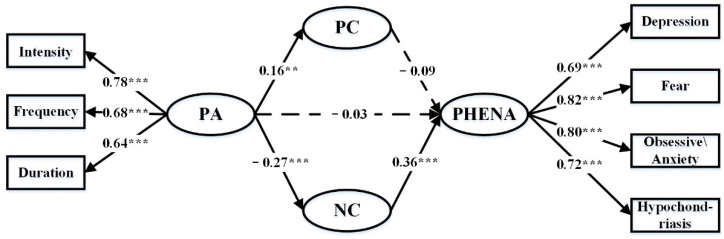
Indirect and mediating effect model. The coefficients in the figure are standardized coefficients. The solid line represents the significant effect of the path, and the dashed line represents the insignificant effect of the path. The ovals and rectangles in this figure do not represent observed variables and latent variables. The ovals are structural variables, and the rectangles are the subdivision dimensions of the structural variable but do not participate in the structural model. *** *p* < 0.001, ** *p* < 0.01.

The final step is to show whether or not the strength of the relationship between PA and PHENA is significantly reduced when PC and NC are added to Model 1. When Model 4 includes the mediator of PC and NC, the influence of PA on PHENA is not statistically significant (β = −0.033, *p* > 0.1), supporting H2. This indicates that the effect of PA is completely mediated by NC. Without considering CS, the direct effect of PA on PHENA is supported by H1. However, while CS was incorporated into Model 1, it is surprising that the effect of PA on PHENA does not seem to have a significant effect. The result provides evidence for the complete mediating role of NC. According to the structural equation model-fitting standard proposed by Bollen [85], all indicators in this study have reached the fitting standard and the model is well fitted. The mediating effect model of CS between PA and PHENA is built as shown in Figure 2. Without considering the mediating effect of CS, the regression coefficient between PA and PHENA is −2.839. After adding CS, the regression coefficient between PA and PHENA is −0.645, PA and PC is 3.015, PA and NC is −4.593, PC and PHENA is −1.775, NC and PHENA is 5.827.

The most popular method to test the mediation effect is the causal steps approach of Baron and Kenny [86]. However, in recent years, the causal steps approach has been criticized and questioned [87,88,89,90]. At present, it is generally believed that the Bootstrap method is a better method to directly test the significance of the coefficient product [90]. The percentile Bootstrap method with offset correction is also used to test the mediating effect of the CS. In the original data, 5000 samples are drawn by repeated random sampling. The results, as shown in Table 3, suggest that the bootstrap 95% confidence interval of the total effect of PA and PHENA contains a value of 0. It indicates that the direct effect of PA on PHENA is not significant. The Bootstrap 95% confidence interval of the indirect effects produced by PC contains a value of 0, which indicates that the mediating effect of PC is not significant as well. On the contrary, the bootstrap 95% confidence interval of the indirect effects produced by NC does not contain a value of 0. It indicates that NC has a significant mediating effect between PA and PHENA. The above results suggest that NC plays a complete mediating role between PA and PHENA, and the mediating effect is 67.83%.

## 4. Discussion

The findings from this study indicate that PA can negatively predict PHENA. The hypothesis H1 is verified, and this extends the previous research conclusions on PA and negative affect [17,22,23]. PA provides people with a kind of emotional regulation or physical confrontation for coping with stress [91], improve the exerciser’s antistress capability [92], and reduce the intensity of stress response [93]. For example, PA can reduce cortisol concentrations during stress, thereby reducing the impact of stress on health [94]. 

The above studies have analyzed the relationship between PA and affect. Some other scholars have also discussed the relationship between PA and coping and the relationship between coping and affect. Most research results support that PA can improve mood, and encourage exercisers to adopt more positive CS and reduce negative CS [95]. CS can affect the nature and intensity of the stress response, and further regulate the relationship between stress and physical and psychological health [96]. This study confirms the mediating role of CS between PA and PHENA, and verifies the validity of hypothesis H2. PA can significantly improve the PC level while reducing the NC level, but only NC can significantly and positively predict PHENA. This result also supports the view that PC and NC have different mediating effects [67,68,69]. 

This study reveals the mechanism between PA and PHENA, with CS playing a mediating role. PA can alleviate PHENA by improving the way of coping with stressful events in the pandemic. CS exerts a completely mediating effect, in which NC plays a mediating role while the PC does not exert a significant mediating effect. The effect of PA on improving CS is probably because PA improves the level of psychological capital appreciation (PCA) and body-esteem of college students [97,98]. Performing moderate-to-high intensity PA can enhance the adolescent’s sense of self-efficacy, which optimizes their CS [77]. PA enables them to have strong confidence in their capability to solve problems and their physical abilities, so that they are more likely to respond to external challenges positively rather than negatively. If more negative CS is used to respond to stressful events, maladaptation may occur, causing individuals to experience higher levels of negative affect [99]. If negative affect is not released in time, it can easily accumulate and finally turn into long-term “chronic diseases” [100]. Furthermore, PA can not only enhance positive mental health but accrue additional health-promoting benefits such as increased immunity among college students [25]. Training regimens during pandemic crises should be introduced as standard habits for health and wellbeing [101]. 

This study has several limitations and areas that can be addressed in future research. First of all, the questionnaire data in this survey were collected in a specific process, so there will be a certain degree of one-sidedness. We recommend longitudinal analysis at different stages of public health emergencies. Secondly, the college students surveyed in this study are all from China’s megacities, so it cannot accurately reflect the situation of college students in other cities in China and abroad. We suggest that it can be expanded to global research for comparison.

## 5. Conclusions

College students’ participation in physical activity can alleviate PHENA through reducing the probability of adopting negative coping mechanisms. The main bodies of the response mechanism for public health emergencies mainly include the government, social organizations and individuals. Reducing the impact of public health emergencies depends on the interaction between the three types of subjects. Therefore, during periods of public health emergency, firstly, the active role of PA in public health emergencies should be brought into full play. PA should be included in the Emergent Public Health Incident Preparedness and Response System. For most developing countries, encouraging PA is a cost-effective way to improve the health of the population. Secondly, attention should be paid to high-risk groups such as college students. The government and educational organizations should play a leading role, while doing a good job in safety education and management, fully mobilizing the individual subjective initiative of college students to participate in PA.

## Figures and Tables

**Table 1:** Descriptive Statistics and Correlations.

Variable	M	SD	Correlations						
			1	2	3	4	5	6	7
1. PA	2.672	0.934	1						
2. PC	3.643	0.734	0.123 **	1					
3. NC	2.677	0.890	−0.186 **	−0.096 *	1				
4. Depression	2.409	0.955	−0.118 **	−0.191 **	0.318 **	1			
5. Fear	2.204	0.995	−0.036	−0.07	0.182 **	0.564 **	1		
6. Obsessive-Anxiety	2.336	0.983	−0.131 **	−0.095 *	0.264**	0.543 **	0.659 **	1	
7. Hypochondriasis	2.688	1.010	−0.117 **	−0.015	0.187 **	0.485 **	0.604 **	0.578 **	1

Note. ** *p* < 0.01, * *p* < 0.05.

**Table 2:** Testing mediator effects using structural equation model.

Testing Steps in Mediation Model	Model 1 Coefficients	Model 2 Coefficients	Model 3 Coefficients	Model 4 Coefficients
Testing Step 1				
Predictor: PA				
Outcomes: PHENA	−0.138 (−2.839) **			
Testing Step 2				
Predictor: PA				
Outcome: PC		0.160 (3.012) **		
NC		−0.272 (−4.593) ***		
Testing Step 3				
Mediator: PC				
Outcome: PHENA			−0.073 (−1.484)	
Mediator: NC				
Outcome: PHENA			0.368 (6.117) ***	
Testing Step 4				
Predictor: PA				
Mediator: PC				0.160 (3.015) **
NC				−0.269 (−4.593) ***
Outcome: PHENA				−0.033 (−0.645)
Overall fit				
χ2/df	1.735	1.781	2.609	2.221
RMSEA	0.033	0.034	0.049	0.042
GFI	0.991	0.985	0.976	0.970
AGFI	0.980	0.974	0.959	0.954
NFI	0.986	0.961	0.955	0.944
IFI	0.994	0.983	0.972	0.968
CFI	0.994	0.982	0.971	0.968
RFI	0.977	0.944	0.936	0.927
RMR	0.028	0.040	0.044	0.046

Note. The coefficients in the table are standardized coefficients. Numbers in parentheses show t-values. *** *p* < 0.001, ** *p* < 0.01.

**Table 3:** Standardized Bootstrap Mediation Effect Test.

Path	Path Effect	SE	Bias-Corrected 95%CI			Percentile 95%CI		
			Lower	Upper	*p*	Lower	Upper	*p*
PA → PHENA	−0.033	0.051	−0.129	0.068	0.555	−0.133	0.064	0.502
PA → PC → PHENA	−0.014	0.011	−0.045	0.001	0.069	−0.038	0.005	0.160
PA → NC → PHE-NA	−0.097	0.028	−0.164	−0.052	0.000	−0.156	−0.047	0.001

Note. Path Effect is the product of all effect values on the path.

## Data Availability

The data presented in this study are available on request from the corresponding author. The data are not publicly available due to confidentiality.
